# Intact Reflexive but Deficient Voluntary Social Orienting in Autism Spectrum Disorder

**DOI:** 10.3389/fnins.2015.00453

**Published:** 2015-12-01

**Authors:** Megan A. Kirchgessner, Alice Z. Chuang, Saumil S. Patel, Anne B. Sereno

**Affiliations:** ^1^Department of Cognitive Sciences, Rice UniversityHouston, TX, USA; ^2^Department of Psychology, Rice UniversityHouston, TX, USA; ^3^Department of Neurobiology and Anatomy, University of Texas Health Science Center at HoustonHouston, TX, USA; ^4^Department of Ophthalmology and Visual Science, University of Texas Health Science Center at HoustonHouston, TX, USA; ^5^Department of Neuroscience, Baylor College of MedicineHouston, TX, USA

**Keywords:** autism, social orienting, reflexive attention, voluntary attention, gaze cueing

## Abstract

Impairment in social interactions is a primary characteristic of people diagnosed with autism spectrum disorder (ASD). Although these individuals tend to orient less to naturalistic social cues than do typically developing (TD) individuals, laboratory experiments testing social orienting in ASD have been inconclusive, possibly because of a failure to fully isolate reflexive (stimulus-driven) and voluntary (goal-directed) social orienting processes. The purpose of the present study was to separately examine potential reflexive and/or voluntary social orienting differences in individuals with ASD relative to TD controls. Subjects (ages 7–14) with high-functioning ASD and a matched control group completed three gaze cueing tasks on an iPad in which individuals briefly saw a face with averted gaze followed by a target after a variable delay. Two tasks were 100% predictive with either all congruent (target appears in gaze direction) or all incongruent (target appears opposite from gaze direction) trials, respectively. Another task was non-predictive with these same trials (half congruent and half incongruent) intermixed randomly. Response times (RTs) to the target were used to calculate reflexive (incongruent condition RT—congruent condition RT) and voluntary (non-predictive condition RT—predictive condition RT) gaze cueing effects. Subjects also completed two additional non-social orienting tasks (ProPoint and AntiPoint). Subjects with ASD demonstrate intact reflexive but deficient voluntary gaze following. Similar results were found in a separate test of non-social orienting. This suggests problems with using social cues, but only in a goal-directed fashion, in our sample of high-functioning individuals with ASD. Such findings may not only explain inconclusive previous findings but more importantly be critical for understanding social dysfunctions in ASD and for developing future interventions.

## Introduction

Social gaze cues are incredibly important for directing our attention toward relevant stimuli and aiding us in our social interactions (Kleinke, 1986; Frischen et al., 2007). Given that autism spectrum disorder (ASD) is characterized by problems with social interactions and communication, it is no surprise that many studies of social attention suggest abnormalities in ASD. For instance, individuals with autism spend less time looking at eyes and more time looking at mouths and objects while viewing live-action movie clips (Klin et al., 2002) and spontaneously follow another person's changing head and eye movements markedly less than typically-developing controls (Leekam et al., 1997). Since social orienting behaviors are particularly important for understanding the subtleties of social interactions, failure to properly engage in social orienting may have serious consequences for everyday social functioning, such as in engaging in joint attention (Dawson et al., 1998), understanding others' intended actions (Castiello, 2003), developing Theory of Mind abilities (Charman et al., 2000), and even acquiring language (Morales et al., 2000; Pruett et al., 2011). Therefore, it is critical to better understand underlying differences in autistic individuals' social orienting capabilities. This knowledge could potentially inform the development of interventions and/or therapies for improving social attention in individuals with autism, which in turn may benefit some of these other key cognitive abilities.

In order to fully grasp the mechanisms underlying social orienting and how these may be abnormal in ASD, it is helpful to first understand how the typically-developing (TD) brain processes and orients attention using non-social stimuli (Posner et al., 1985). Non-social orienting can be thought of in terms of two distinct processes: reflexive (automatic, stimulus-driven) orienting and voluntary (goal-directed) orienting. According to Sereno (1992)'s Tonic Inhibition Model (TIM), voluntary orienting is largely governed by the prefrontal cortex, which, via the basal ganglia, modulates the superior colliculus (thought to be important for reflexive orienting). Consequently, the ability to resist a reflexive response toward an illuminated peripheral stimulus, such as in an antisaccade task, requires additional inhibition of the reflexive orienting system by the prefrontal cortex (i.e., the voluntary system; Munoz and Everling, 2004). An impairment or loss of this inhibition could result in faster response times (or more impulsive responding) in the direction of the stimulus as well as slower (and fewer) responses to the opposite direction because a response in the direction opposite from the stimulus requires sufficient voluntary control.

Some have wondered whether social and non-social orienting processes are unique and whether a reflexive and voluntary distinction is also present in social orienting. Similar social reflexive facilitation effects (i.e., faster responses to cued than to non-cued locations) and voluntary facilitation effects (i.e., faster responses to predicted locations) as have been robustly shown using peripheral non-social cues (e.g., Posner et al., 1985) have also been shown using social cues (Friesen and Kingstone, 1998; Friesen et al., 2004). Nevertheless, there are distinct differences between social and non-social orienting processes. For instance, the well-established inhibition of return (IOR) effect in non-social reflexive orienting tasks, whereby subjects become *slower* at responding to cued than non-cued locations when the time between the cue and target is longer than 300 ms (Posner et al., 1985), appears to be absent in social orienting tasks (Friesen and Kingstone, 1998). This could be because the superior temporal sulcus (STS) is specifically recruited for reflexive *social* orienting instead of the superior colliculus for *non-social* orienting (Friesen and Kingstone, 1998). The importance of the STS for only *social* orienting is supported by a neuropsychological case study of a patient with a lesion to the right STS who demonstrated the well-established reflexive facilitation effect to non-predictive, non-social stimuli, but not to social stimuli (Akiyama et al., 2006). This dissociation strongly suggests that the social orienting system is not identical to the non-social orienting system.

Although social and non-social orienting involve separable systems, social orienting appears to be similarly divided into reflexive and voluntary orienting processes. Hill et al. (2010) used a social gaze cueing paradigm to demonstrate that orienting to social stimuli involves divisible reflexive and voluntary processes, analogous to non-social orienting. By varying the amount of time between the onset of a face and a target stimulus (stimulus onset asynchrony, SOA) and whether the gaze cue was predictive of the target location, they were able to show the time courses of the separable reflexive and voluntary social orienting systems. In particular, they demonstrated a facilitation effect for congruent (i.e., gaze direction corresponds to target location) relative to incongruent trials when the SOA was short, which is indicative of reflexive social orienting. When the congruency of the trials was held constant and the predictive value of the gaze cue was examined, the predictive cues resulted in response facilitation relative to non-predictive cues when the SOA was longer (200 ms or more). This slower onset of response facilitation for predictive trials represents voluntary control. Therefore, their experiments were very important for demonstrating two distinct yet interacting processes involved in orienting to social stimuli in adult subjects.

Since autistic individuals orient less to social cues such as eyes and facial expressions than do typical individuals in many contexts (Dawson et al., 1998; Klin et al., 2002; Guillon et al., 2014), many studies have attempted to look at whether people with ASD show social attention deficits. Although orienting based on central non-social cues seems intact (Senju et al., 2004; Greene et al., 2011; Pruett et al., 2011), many studies have been inconclusive and have produced conflicting results with regards to social orienting (see Nation and Penny, 2008; Landry and Parker, 2013 for reviews). Some studies have found a lack of reflexive orienting to social gaze cues in children with autism (Ristic et al., 2005; Goldberg et al., 2008; Gillespie-Lynch et al., 2013), while others have found no differences from TD children (Chawarska et al., 2003; Swettenham et al., 2003; Kylliäinen and Hietanen, 2004; Senju et al., 2004; Greene et al., 2011; Pruett et al., 2011). It is important to note that none of these studies utilized and truly separated reflexive and voluntary conditions. In all of these studies that used a “predictive,” or voluntary, condition (Senju et al., 2004; Ristic et al., 2005; Pruett et al., 2011), those conditions were only 80% predictive and oftentimes included a stimulus-driven or reflexive cueing component. Consequently, these measurements of voluntary cue effects may still be confounded by reflexive orienting influences, thus complicating interpretation of results. Since social attention can be broken down into reflexive and voluntary orienting processes, and given conflicting findings in laboratory studies of social orienting in ASD, it is feasible that autistic individuals' impairments are specific to a certain kind of attention.

In particular, it is possible that the voluntary, but not the reflexive, aspect of social orienting is selectively impaired in ASD. Since most of these studies have focused primarily on reflexive orienting and found no differences in ASD, it is feasible that reflexive social orienting truly is intact in ASD. Conversely, a few studies have raised the possibility that specifically voluntary social orienting is deficient (Senju et al., 2004; Hill et al., 2010). This is supported by the fact that Klin et al. (2002) reported that there was no correlation between amount of time spent fixating on eyes and social competence. This might suggest that individuals with ASD do not find the eyes particularly helpful or meaningful (Klin et al., 2002), and this lack of correlation may be closely related to the more *voluntary* aspects of social attention. If voluntary social orienting is selectively impaired, this may explain why many of the previously mentioned studies have found no differences in social orienting. Since subtle deficits in voluntary orienting may have been washed out or masked by normal or even hyper reflexive orienting effects, this might also help explain why many more naturalistic studies have found social orienting differences in ASD (Greene et al., 2011). These types of studies may be inherently more context-dependent, relying more heavily on voluntary orienting processes.

Therefore, it is important to develop a method that can adequately probe each of these orienting processes while controlling for the other to see whether one, but not the other, is impaired in ASD. Adapting Hill et al. (2010) paradigm for a tablet may be a promising approach because a tablet-based approach has recently been shown to be an effective tool for measuring reflexive versus voluntary non-social orienting (Zhang et al., 2013). Since tablets are simple, portable, less intimidating for children, and easier to operate, they may be especially useful for measuring social orienting differences in the ASD population. Moreover, a tablet-based application is an especially promising avenue for the development of future interventions. If children with autism are able to reflexively orient to social stimuli, it may be feasible to develop tablet-based applications to train them to use that social information in a more goal-directed manner, i.e., improve their voluntary social orienting and/or responses to particular social expressions and gaze signals. Importantly, a social cueing task like the one already mentioned may provide the basis for such a training method, especially if developed in a simple gaming format on an accessible, portable, and easy-to-use platform, such as a tablet device.

Thus, the current study has two primary aims. First, we investigate differences in reflexive and voluntary social orienting in children with ASD, and we hypothesize that these individuals will be selectively impaired in voluntary social orienting but not reflexive social orienting. Second, we hope to establish the tablet as a viable means of measuring reflexive and voluntary social cueing effects in this and potentially other clinical populations. We hope that this work will improve our understanding of social differences in ASD and pave the way for the development of tablet-based interventions to target specific social attention deficits.

## Experiment 1

In this first experiment, we use an application on an iPad 2 to measure reflexive versus voluntary social orienting in children with and without high-functioning ASD. We aim to improve upon previous social orienting experimental designs by having 100 and 50% predictive conditions each with the same congruent and incongruent trials, allowing us to separately examine the effect of congruency (reflexive orienting) while keeping predictability constant, and predictability (voluntary orienting) while keeping congruency constant.

### Materials and methods

#### Participants

The study was approved by The University of Texas Health Science Center Committee for the Protection of Human Subjects and was in accordance with the Declaration of Helsinki. Before participating, all subjects provided their informed assent with parental consent and were debriefed following their participation. High-functioning ASD subjects were recruited from two schools around the Houston area that specialize in high-functioning autism. Typically developing (TD) control subjects were recruited by word of mouth and approved flyers and matched on age and gender as closely as possible to the ASD subjects. None of the control subjects had ever received an ASD diagnosis. With the exception of two subjects, ASD diagnosis was confirmed with a questionnaire conducted over the phone (8), authenticated with medical records at the school (3), or both (5). Phone interviews included questions regarding the age at which symptoms were first noticed, when was the date of ASD diagnosis, how old was the subject when diagnosed, details of the diagnosis (e.g., Asperger's or PDD-NOS), and the name of the doctor and clinic that issued the diagnosis. Of 18 recruited ASD subjects, 3 were excluded from further analysis whose medical records did not indicate any ASD diagnosis. One additional subject was excluded due to a confounding condition of severe language impairment and a low verbal subscore on the Wechsler Abbreviated Scale of Intelligence (WASI-III; Wechsler, 1991), more than two standard deviations outside of the normal range. The remaining subjects included 15 TD controls [mean age = 9.13 (±1.96) years old, 6 (40%) girls] and 14 subjects with ASD [mean age = 9.57 (±2.10) years old, 2 (14%) girls].

#### Stimuli

An application for the iPad 2 with tasks based on the Hill et al. (2010) study was developed in the lab and used to conduct the experiment. This application contains three social cueing tasks. In each of these tasks, there is a solid white circle (diameter = 1.4 cm) that first appears in the center of the dark iPad screen that serves as a finger fixation point. Four different gray scale face images from two female individuals act as the gaze cues, each with gaze averted 45 degrees to the right and left (face stimuli identical to Hill et al., 2010). The target is a solid white square (1 cm) presented to the left or the right of the finger fixation point. A trial is initiated by touching and holding the finger fixation point, and after a delay (833 ms), a face briefly appears for 17 ms. Then, the target is presented to the left or the right at one of five stimulus onset asynchronies (SOAs): 25, 58, 174, 523, or 805 ms. These SOAs were chosen so that we would have two shorter SOAs to see reflexive cue effects (which are typically early and short-lived), one intermediate point, and two longer SOAs so as to see voluntary cue effects (which should emerge around 200 ms). The target remains on screen until the screen is re-touched. Response locations and times of finger lift off were recorded using the iPad 2's capacitive screen with a spatial resolution of 52 pixels per cm and a frame refresh rate of 60 Hz. Response times were adjusted by subtracting the externally-measured (with photodiode, microphone and storage oscilloscope; see Sereno et al., 2014) average delay of detecting a touch event on the capacitive device (73.88 ms). The SD of the delay was approximately 5.9 ms. Comparing this variability to the mean RT (on average 353.5 ms), the coefficient of variation (CV = SD/mean) was less than 2%. In addition, this variation in the delay is less than 15% of the total variation in RT (SD, on average about 41.1 ms), including between subjects variation. Hence, this variability was well below the RT variations in individual human performance we measured.

#### Study design

**Figure 1:**
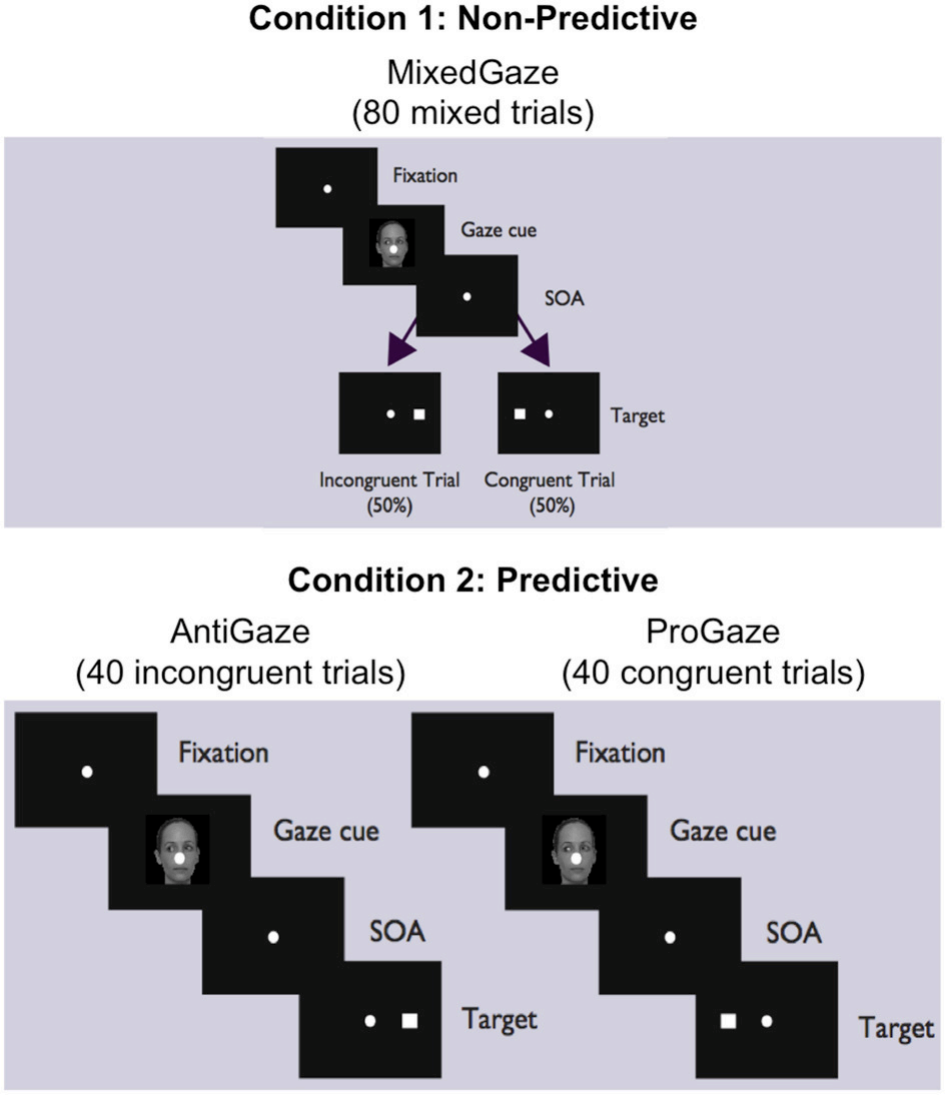
**MixedGaze, AntiGaze, and ProGaze tablet-based gaze-cueing tasks**. A schematic of our two conditions (Condition 1: Non-predictive; Condition 2: Predictive) and three social gaze-cueing tasks (MixedGaze, AntiGaze, and ProGaze). Each condition contained the same 80 trials, half of which were congruent (i.e., target appears in same direction as gaze) and half of which were incongruent (i.e., target appears in opposite direction from gaze).

The experiment consisted of two conditions (Figure 1). In Condition 1 (Non-predictive condition, i.e., MixedGaze, or MG), the target appeared in the same direction as the gaze cue (congruent) in half of the trials and in the opposite direction (incongruent) in the other half; therefore, the gaze cue was not informative (i.e., 50% predictive). Condition 1 consisted of 80 trials (2 congruencies × 5 SOAs × 4 cue images × 2 repetitions). In Condition 2 (Predictive condition), gaze cues were always 100% predictive of the location of the target. Condition 2 contained two subtasks: ProGaze (PG), in which the target always appeared in the same direction as the gaze cue, and AntiGaze (AG), in which the target always appeared in the opposite direction. Each subtask consisted of 40 trials (5 SOAs × 4 cue images × 2 repetitions). The combined 80 trials in the two subtasks of Condition 2 were identical to the 80 trials used in Condition 1. The order of conditions, as well as the order of Condition 2 subtasks (always kept together), was counterbalanced across participants, creating four possible task orders.

In this experiment, Group (TD Control vs. ASD) was the between-subject independent variable, and Congruency (Congruent vs. Incongruent), Predictability (Predictive vs. Non-predictive), and SOA (25, 58, 174, 523, 805 ms) were within-subject independent variables. The dependent variable was response time (RT), measured as the time from when the target is presented to when the subject initiates their response by lifting their finger from the center fixation point. The number of errors was also recorded, but, given very few errors (0.99 and 3.03% for Control and ASD groups, respectively), they were not analyzed.

#### Procedure

TD Control subjects were tested in a darkened testing room at the University of Texas Medical School in Houston (UTMSH). The TD control subjects and their parents were compensated with $20 for their time and for travel/parking costs, and the children were also awarded with small toys. High-functioning autistic subjects were recruited from two schools around the Houston area that specialize in high-functioning autism, and they were tested directly at their schools in a darkened testing room with an environment mimicking the Medical School testing room. Because there was no parental travel/parking costs involved, these children were not compensated monetarily but were instead awarded with toys following their participation.

All subjects were first given instructions and allowed to practice a few (4–6) trials for each of the tasks in the same order that they would complete the experiment. This was to generally acquaint them with the iPad and the overall tasks so that they could see the differences between the subtasks and so that they were able to ask questions and have ample practice with each one. In the Condition 1 task (MG), subjects were told that sometimes the target would appear in the same direction that the eyes were looking, and other times it would appear away from where the eyes were looking. Therefore, it was emphasized that the eyes would *not* tell them where the target would appear. For the Condition 2 tasks, subjects were told that the target would always appear in the same direction (PG subtask) or opposite direction (AG subtask) from where the eyes of the face were looking. For each condition and subtask, they were told to touch the target square as quickly but as accurately as possible.

Before performing each task, subjects were prompted to recall that task's instructions (e.g., PG: In this game, will the eyes of the face be looking toward the square or away from the square?), and corrected if they answered incorrectly. This was to ensure that they understood the task-specific instructions. Then, they were given a few more practice trials to make sure they had sufficient practice and understood the task before the full experimental data was collected.

Verbal and Matrix reasoning subtests from the Wechsler Abbreviated Scale of Intelligence (WASI-III) were also administered to all ASD subjects (Wechsler, 1991). The estimated IQ scores from the two WASI subtasks ranged from 80-133 with a mean (±SD) of 100.71 (±17.18), suggesting no difference in IQ [*t*_(13)_ = 0.2, *p* = 0.9] from the average IQ score (100) of age-matched TD children (Wechsler, 1991).

#### Data analysis

A MATLAB script was used to extract data and trim errors and outliers. Responses were considered an “error” if the distance between target location and iPad-calculated location of subject's response was greater than 3.3° (1.9 cm). The error rate for the TD Control group was 0.99% (PG: 1.15%, AG: 0.66%, MG: 1.07%), and for the ASD group it was 3.03% (PG: 3.11%, AG: 3.11%, MG: 2.95%). Once these error trials were excluded, additional trials were filtered out from the final analysis if their RT was less than 150 ms, greater than 800 ms, or more than 2.5 standard deviations away from their mean RT for that particular SOA. In addition, we removed trials where the subject lifted their finger but did not respond in a timely fashion (i.e., duration of movement greater than 2.5 standard deviations from the mean movement duration for that particular SOA). Together, these trimming procedures resulted in the removal of 3.08% of non-error trials (PG: 3%, AG: 3.17%, MG: 3.08%) in the control group and 9.46% (PG: 8.39%, AG: 9.64%, MG: 9.91%) in the ASD group.

Once error trials were removed and the data was filtered, each subject's remaining RTs comprised the set of observations for each of the five SOAs and each of four different *Trial Types* (Figure 1): MG congruent (Condition 1, right), MG incongruent (Condition 1, left), PG (congruent; Condition 2, right panel), and AG (incongruent; Condition 2, left panel), trials. These four Trial Types were used to evaluate the following four *Cue Effects*:

1. Reflexive orienting in non-predictive (NP) condition (Condition 1) = MG Incongruent RT vs. MG Congruent RT.
2. Reflexive orienting in predictive (P) condition (Condition 2) = AG (Incongruent) RT vs. PG (Congruent) RT.
3. Voluntary orienting in congruent trials = MG Congruent RT (Condition 1, right) vs. PG RT (Condition 2, right panel).
4. Voluntary orienting in incongruent trials = MG Incongruent RT (Condition 1, left) vs. AG RT (Condition 2, left panel).

Given there were repeated measures in each subject and different variability between groups and among SOAs, a mixed effect analysis was first performed on RT for each group and each of 5 SOAs to examine Cue Effects among the four different Trial Types (PG congruent, AG incongruent, MG congruent, and MG incongruent). In this mixed effect model, Trial Type is both a fixed effect and a random effect with compound symmetric covariance structure, and Subject is a random effect with an autoregressive order 1 covariance structure.

To examine group differences in Cue Effects, we performed a second mixed effect analysis. In these analyses, the fixed effect was Group (TD Control and ASD), and both Trial Type and Subject were the random effects with the same covariance structure as in the first analysis. Again, Cue Effects between Groups were compared at each SOA.

Since each subject only completed 8 of each trial type for each SOA, combining some of the SOAs that were expected to be measuring the same process simplifies the presentation but does not change the findings we report. Hence we repeated the above analyses after combining the two early SOAs (25 and 58 ms) and the two late SOAs (523 and 805 ms) to make three levels of delay: short (25 and 58 ms), intermediate (174 ms), and long (523 and 805 ms). These collapsed analyses (with 3 levels of delay) are presented below in the Results, and estimated means and mean differences from these analyses are used in the Figures. Estimated means (±SE), cue effects, and significance levels for the collapsed delays analyses are presented in Supplementary Tables 1, 2. A *p* < 0.05 was considered as statistically significant.

### Results

#### Reflexive orienting in non-predictive (NP) condition

**Figure 2:**
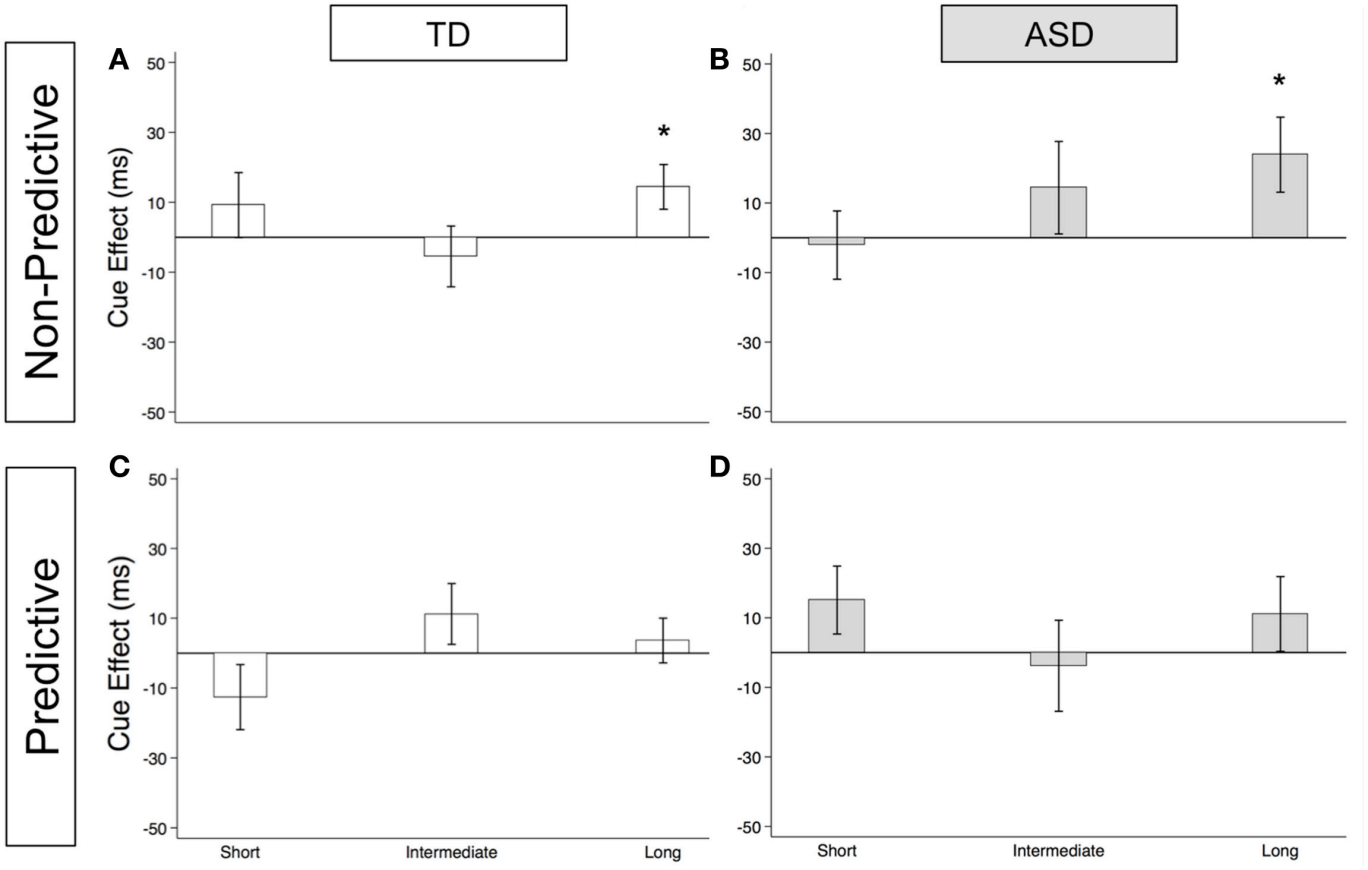
**Estimated mean reflexive social orienting cue effects in TD control and ASD children**. Estimated mean reflexive cue effects in TD controls (first column; **A,C**) and subjects with ASD (second column; **B**,**D**) in non-predictive (top row; **A**,**B**) and predictive (bottom row; **C**,**D**) conditions. A star indicates a significant cue effect (^*^p < 0.05). Error bars represent one standard error.

**Figure 3:**
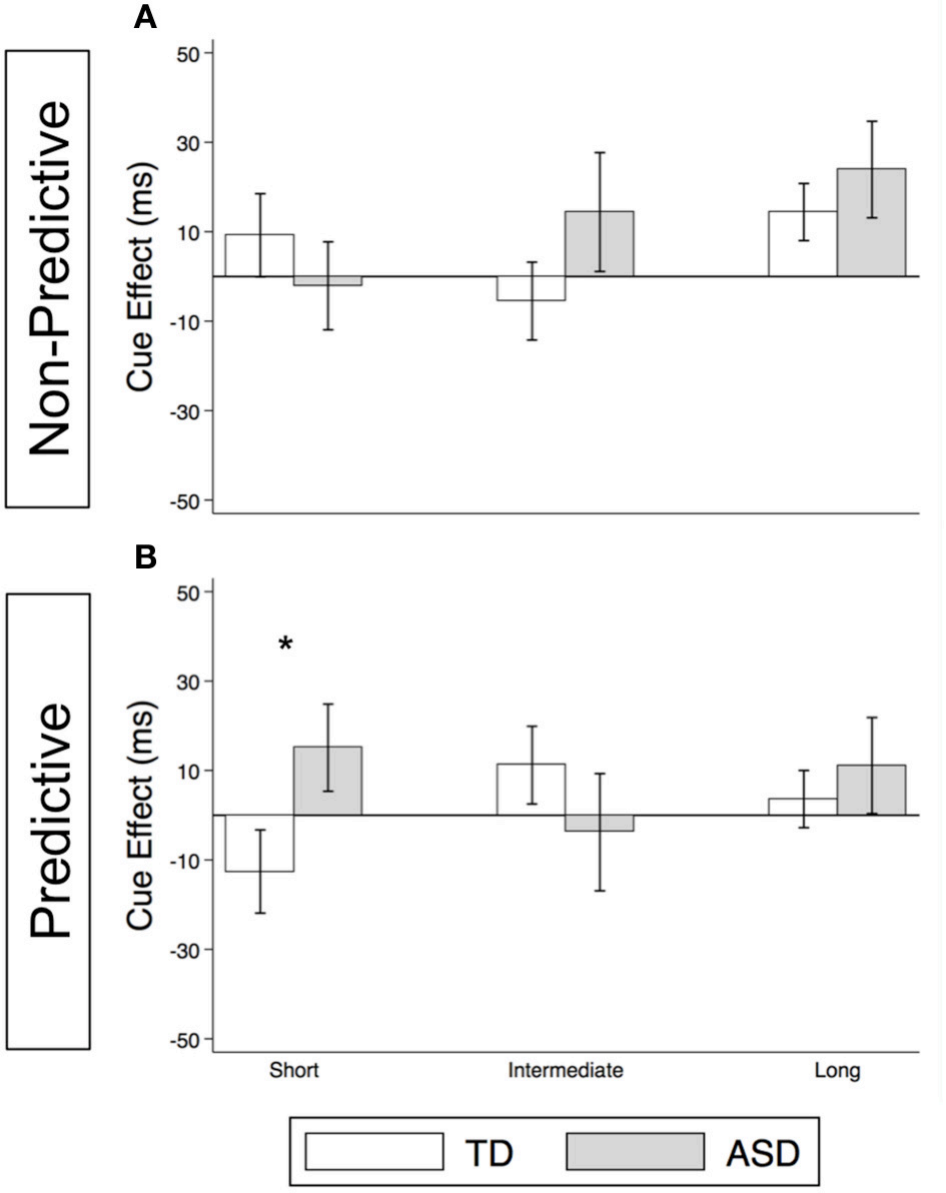
**Reflexive social orienting is intact or enhanced in children with ASD relative to TD controls**. Comparison of estimated mean reflexive cue effects between TD control (white) and ASD (gray) groups in non-predictive **(A)** and predictive **(B)** conditions. Estimated mean cue effects and error bars are the same as in Figure 2. A star indicates a significant group difference (^*^p < 0.05).

As illustrated in Figures 2A (TD) and 2B (ASD) and in Supplementary Table 1, both groups show significant reflexive facilitation at the long delay [TD: 14.4 ms (± 6.4), *t*_(56)_ = 2.3, *p* = 0.028; ASD: 23.9 ms (± 10.8), *t*_(52)_ = 2.2, *p* = 0.031]. In other words, by about 523 ms, both TD and ASD subjects are faster at congruent than incongruent trials even though the gaze cue is not predictive of the target location. This finding at the long delay is consistent with the findings at SOA of 523 ms in the uncollapsed data [TD: 20.4 ms (± 8.2), *t*_(56)_ = 2.5, *p* = 0.016; ASD: 34.5 ms (± 16.0), *t*_(52)_ = 2.2, *p* = 0.036]. Although the ASD group shows greater reflexive cue effects at the long delay [Group difference = 9.6 ms (± 12.3)], none of these Group differences reach statistical significance (Figure 3A; *p*'s > 0.2).

#### Reflexive orienting in predictive (P) condition

As illustrated in Figures 2C (TD) and 2D (ASD) and in Supplementary Table 1, neither group shows significant reflexive facilitation at any of the three delays (*p*'s > 0.1). However, reflexive facilitation at the short delay is greater in the ASD than in the TD control group, and this difference is significant [Figure 3B; Group difference = 28.0 ms (± 13.5), *t*_(1675)_ = 2.1, *p* = 0.039]. From the uncollapsed data, it appears that this group difference emerges at the shortest SOA of 25 ms [Group difference = 49.1 ms (± 16.2), *t*_(780)_ = 3.0, *p* = 0.003] and disappears by SOA of 58 ms [Group difference = 5.0 ms (± 17.6), *p* = 0.8].

#### Voluntary orienting in congruent trials

**Figure 4:**
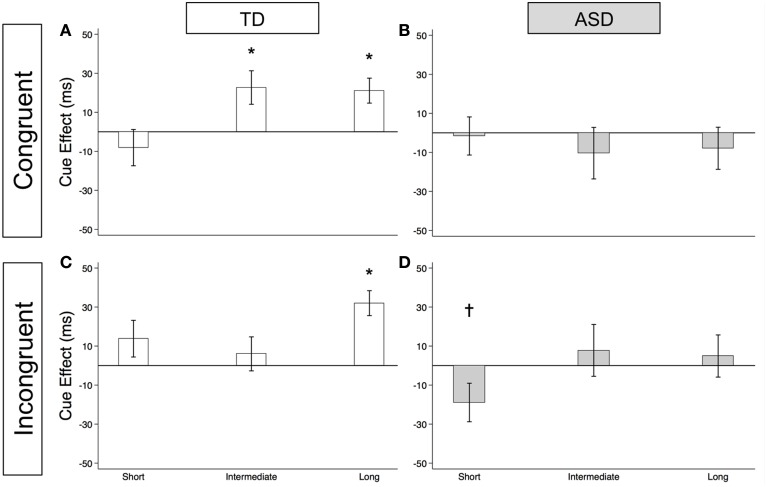
**Estimated mean voluntary social orienting cue effects in TD control and ASD children**. Estimated mean voluntary cue effects in TD controls (first column; **A,C**) and subjects with ASD (second column; **B**,**D**) in congruent (top row; **A**,**B**) and incongruent (bottom row; **C**,**D**) trials. A star indicates a significant cue effect (^*^p < 0.05) and a cross indicates a marginally significant cue effect (^†^*p* < 0.10). Error bars represent one standard error.

**Figure 5:**
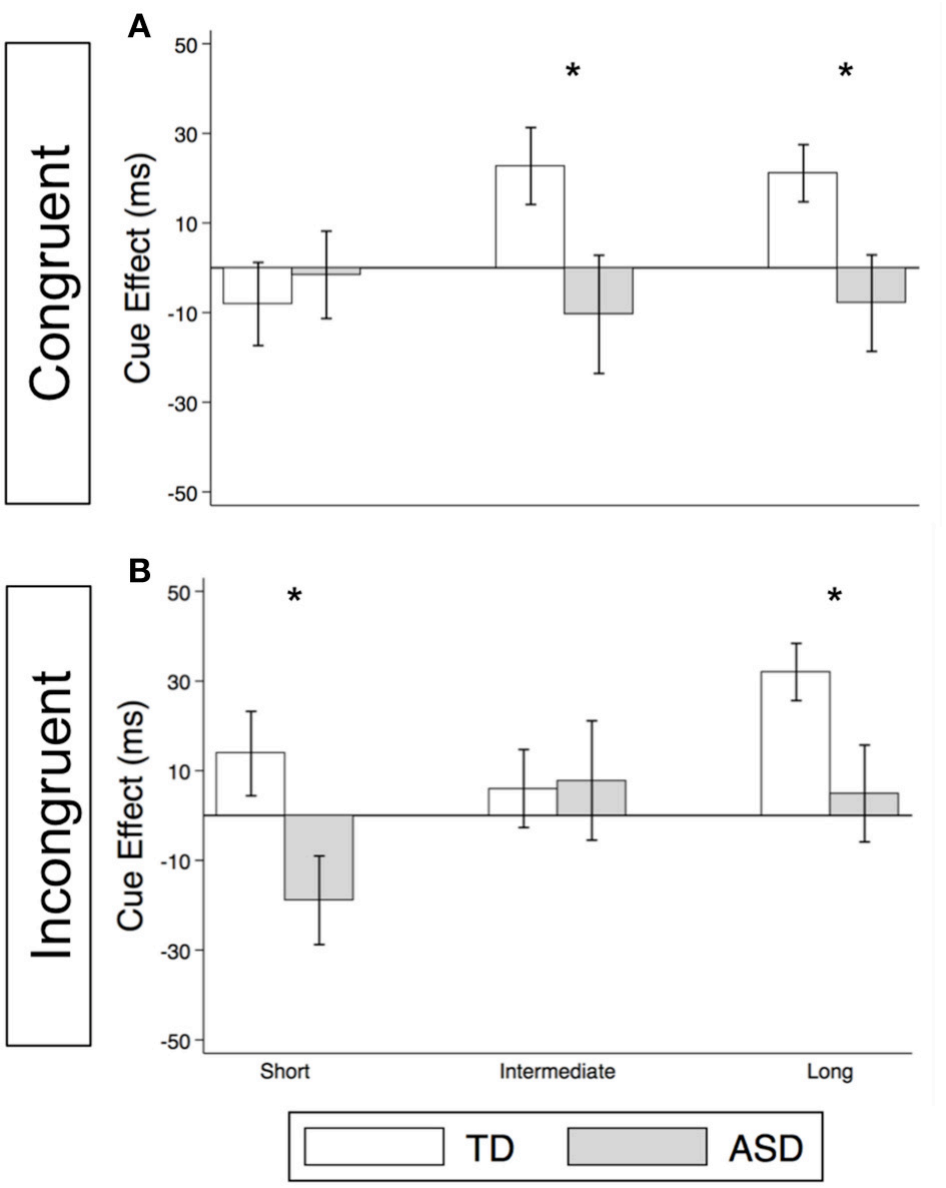
**Voluntary social orienting is deficient in children with ASD relative to TD controls**. Comparison of estimated mean voluntary cue effects between TD control (white) and ASD (gray) groups in congruent **(A)** and incongruent **(B)** trials. Estimated mean cue effects and error bars are the same as in Figure 4. A star indicates a significant group difference (^*^p < 0.05).

As illustrated in Figure 4A and Supplementary Table 2, the TD Control group shows a significant voluntary cue effect at both the intermediate [22.7 ms (± 8.6), *t*_(56)_ = 2.6, *p* = 0.011] and long delays [21.1 ms (± 6.4), *t*_(56)_ = 3.3, *p* = 0.002]. This was also true of the uncollapsed data for the intermediate (same as collapsed data) and long SOAs [SOA = 523 ms: 14.4 ms (± 8.3), *t*_(56)_ = 1.7, *p* = 0.087; SOA = 805 ms: 28.0 ms (± 8.8), *t*_(56)_ = 3.2, *p* = 0.002], although the cue effect at SOA = 523 ms was marginal. The positive cue effects in the control group at these longer delays reflect the advantage of predictive over non-predictive trials and suggest that in TD children this first becomes significant at an SOA of 174 ms. In the uncollapsed data in the TD Control group, we find a negative cue effect at the shortest SOA [−21.3 ms (± 10.4), *t*_(56)_ = −2.1, *p* = 0.045], similar to previous work in normal adults with this paradigm (Hill et al., 2010); however this cue effect is not significant in the collapsed data at the short delay (Figure 4A; *p* = 0.4). In contrast, there are no significant cue effects in the ASD group (Figure 4B, *p*'s > 0.4), meaning that ASD children's response times are about the same in the P and NP conditions and thus they show no indication of using the predictive cues. The only effect that approaches significance is a marginal *negative* cue effect at SOA = 523 ms in the uncollapsed data [−27.9 ms (± 16.1), *t*_(52)_ = −1.7, *p* = 0.089], suggesting that if anything, ASD subjects are *slower* when the gaze cue has predictive value. Further, voluntary facilitation is significantly greater in the TD control than in the ASD group at both the intermediate [Group difference = 33.0 ms (± 15.4), *t*(774) = 2.1, *p* = 0.033] and long delays [Group difference = 29.1 ms (± 12.3), *t*_(1651)_ = 2.4, *p* = 0.018; Figure 5A].

#### Voluntary orienting in incongruent trials

For this voluntary cueing condition, as illustrated in Figures 4C (TD) and 4D (ASD) as well as in Supplementary Table 2, the findings are similar in that there are significant voluntary cue effects at the long delay for the TD control group [32.0 ms (±6.4), *t*_(56)_ = 5.0, *p* < 0.001] but not for the ASD group at either the intermediate or long delays [*p*'s > 0.5]. Furthermore, voluntary facilitation at the long delay is significantly greater in the TD than in the ASD group [Group difference = 27.2 ms (±12.3), *t*_(1651)_ = 2.2, *p* = 0.027; Figure 5B]. There are a few small differences in this incongruent Voluntary condition from the congruent Voluntary condition. Namely, in the TD control group in the incongruent Voluntary condition (Figure 4C), significant voluntary cue effects are not significant at the intermediate SOA but emerge at slightly longer delays [also true in the uncollapsed data, SOAs of 174 ms: 6.0 ms (± 8.7), *p* = 0.5; vs. 523 ms: 27.0 ms (± 8.3), *t*_(56)_ = 3.2, *p* = 0.002]. Given the lack of a significant voluntary cue effect in the TD group at the intermediate SOA, there are also no significant group differences at this SOA (Figure 5B). Another difference from the congruent trials is that in the ASD group, there is a marginally significant negative cue effect at the short delay [−18.9 ms (± 9.9), *t*_(52)_ = −1.9, *p* = 0.062; Figure 4D], and this results in a significant group difference at the short delay [32.9 ms (± 13.7), *t*_(1675)_ = 2.4, *p* = 0.016; Figure 5B].

### Discussion

#### Differences between non-predictive and predictive reflexive social orienting cue effects

First, our findings demonstrate intact reflexive social orienting in children with ASD. Like the TD control group, they were consistently faster in congruent than in incongruent trials of the non-predictive (NP) condition after 25 ms, and this facilitory reflexive cue effect reached significance at the long delays (>523 ms; Figures 2A,B). Further, there was some evidence of heightened reflexive social orienting in individuals with ASD. Namely, there was a significant group difference with greater early facilitation demonstrated by the ASD subjects in the predictive (P) condition (Figure 3B). Although there were no significant group differences in the NP condition (Figure 3A), in agreement with the P condition finding, cue effects by the ASD subjects in the NP condition were consistently larger than in the control group at intermediate and long delays, albeit non-significantly. Thus, taken together, our findings suggest reflexive social orienting is intact or even enhanced in ASD individuals. Although to our knowledge no previous studies have argued for enhanced reflexive social orienting in children with ASD, Senju et al. (2004) did find that their ASD group showed a greater cue validity effect (i.e., faster responses on congruent than incongruent trials) than the control group in a non-predictive gaze cueing task. Further research is needed to determine whether this aspect of social orienting is truly *enhanced*. Still, children with ASD strongly demonstrated reflexive social orienting, and therefore this aspect of attention appears to be intact in this group.

Secondly, the time course of reflexive cue effects differed in NP and P conditions with significant facilitation occurring for both groups at the long delay in the NP condition (Figures 2A,B), but not in the P condition (Figures 2C,D). Based on previous work (Hill et al., 2010), we did expect there to be a difference in P and NP reflexive cue effects at the later SOAs. That is, in the P condition, where subjects know where the target is going to appear, we expected any automatic reflexively-generated cueing differences between a congruent and incongruent cue to go away with the growing influence of voluntary control. When this happens, voluntary control directs (or re-directs) attention to the correct upcoming target location, enabling a faster response in both the PG and AG subtasks and thus reducing the reflexive facilitory cue effect to zero at long SOAs. On the other hand, in the NP condition, where subjects understand that the cue is meaningless in predicting upcoming target location, we have previously reported that a social cue does not produce inhibition of return at longer SOAs but rather continues to show evidence of reflexive facilitation at 500 and 750 ms (Hill et al., 2010). Consistent with the previous adult findings, both groups demonstrated significant reflexive facilitation at long (523 and 805 ms) delays in the NP condition (Figures 2A,B).

There were some unanticipated findings at the early delays where we expected to find reflexive social facilitation. Most importantly, neither group showed significant facilitation at short SOAs in the NP condition (Figures 2A,B). Specifically, although the TD group did show a nonsignificant positive cue effect (at the short delay in the collapsed data: 9.2 ms), the ASD group hovered close to 0 (−2.1 ms). Additionally, in the P condition, we observed a nonsignificant negative reflexive cue effect in our control group at the short delay (−12.6 ms, Figure 2C), whereas the ASD group showed a nonsignificant positive effect (15.1 ms, Figure 2D). A negative cue effect in the TD control group means that these subjects were responding faster on AG compared to PG trials at short SOAs. This was unexpected based on previous findings reported by Hill et al. (2010) and our own data from typical adults who completed these same tablet-based social gaze tasks (unpublished data); in both cases, we have observed significant *positive* cue effects at the earliest delays which diminish around 200 ms. It is possible that this negative cue effect in TD Control participants was the result of these young subjects' perception of the AG task as a tricky or “harder” task, leading to an increased level of arousal on the AG trials (faster RTs) that shifted this difference curve down. Given control subjects were tested in a lab setting in a Medical School whereas the ASD children were tested within a testing room within their own school, it is also possible that control children were more cautious of the testing instructions in an unfamiliar location.

#### Lack of evidence of voluntary social gaze cueing in ASD

Meanwhile, although we observed robust voluntary social orienting effects in our control group after 174 ms, the ASD group did not show any such effects (Figures 4, 5). Starting around 174 ms, TD controls demonstrated consistently significant positive voluntary cue effects in both congruent and incongruent trials (Figures 4A,C); in other words, given time for voluntary control to become operational and enable the advantageous use of the gaze cue, TD participants became significantly faster at P than NP conditions. However, the ASD group differed from the TD control group in that they did not show any of these effects (Figures 4B,D). These group differences (Figure 5) suggest that the ASD subjects are not using the predictive gaze cues to their advantage, despite having been explicitly told that the gaze cue will help them locate and thus respond faster to the target in the P condition. In sum, the TD control group demonstrates clear use of voluntary control in social orienting while the ASD group does not.

#### Broader deficits?: Social and non-social orienting

However, an alternative possible interpretation of our findings in Experiment 1 is that these differences in voluntary social orienting are not *social, per se*, but rather reflect broader executive function deficits that affect both social and non-social voluntary orienting (see Hill, 2004 for a review). That is, deficits in the voluntary social gaze orienting tasks could reflect a broader problem in voluntary orienting. The antisaccade task, which is a traditional test of non-social voluntary orienting, is often used to measure more general problems with executive functions (Sereno et al., 2009). A recent tablet-based version of the antisaccade task (“AntiPoint”) that measures touch responses rather than eye movements has also been used to measure executive dysfunction (Zhang et al., 2013). If high functioning ASD participants have a broader deficit in executive function, they should demonstrate dysfunction on the AntiPoint task as well. However, many studies have found that children with autism are not uniformly impaired in all realms of executive functioning (Griffith et al., 1999; Liss et al., 2001; Geurts et al., 2004). For instance, Liss et al. (2001) investigated executive functions in TD children, children with developmental language disorder, and children with high-functioning ASD using a number of tests. The only observed differences were on measures of perseveration in the ASD group, but even these differences disappeared when verbal IQ was used as a covariate. Thus, we hypothesized that we would not detect any differences between the TD control and high-functioning ASD groups on either ProPoint (reflexive orienting) or AntiPoint (voluntary orienting) tasks, suggesting that our findings in Experiment 1 indicate differences in voluntary orienting specific to *social* processes rather than a more general deficit in executive functioning.

## Experiment 2

In this experiment, we tested the same two groups of subjects on two additional non-social orienting tasks: ProPoint and AntiPoint. We hypothesized that high-functioning ASD subjects would not differ from control subjects in either the ProPoint or AntiPoint task, suggesting the findings in Experiment 1 with our population of ASD subjects do not indicate a more generalized voluntary orienting deficit.

### Materials and methods

#### Participants

All control subjects (15) and 13 of the 14 ASD subjects who participated in Experiment 1 also participated in Experiment 2.

#### Stimuli

In these tasks, the central fixation point is the same as in the social tasks but is surrounded by four square boxes, each 4 cm from the central fixation point. These boxes remain on screen throughout the duration of the task, and they indicate the possible locations of the targets. The target (white square, 0.8 cm) appears in any of the four target locations 480 ms after the fixation point is touched.

#### Study design

**Figure 6:**
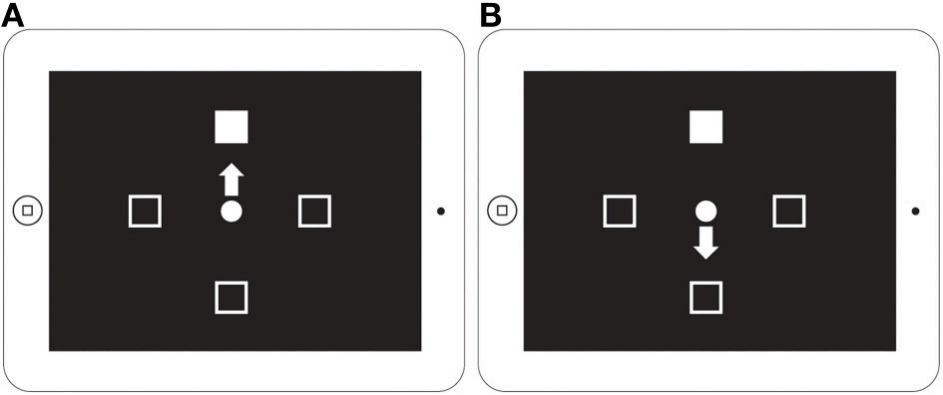
**ProPoint and AntiPoint non-social orienting tasks displayed on iPad screen**. A schematic of the ProPoint **(A)** and AntiPoint **(B)** non-social orienting tasks used in Experiment 2 (figure from Zhang et al., 2013). ProPoint **(A)** is a test of reflexive orienting while AntiPoint **(B)** is a test of voluntary orienting.

Experiment 2 consists of two subtasks and uses the same design as was used in the Zhang et al. (2013) study (Figure 6). The ProPoint (PP) task is a test of reflexive orienting because it requires the subject to simply touch the response box containing the target. The AntiPoint (AP) task is a test of voluntary orienting. In this task, the subject must inhibit their reflexive response to touch the box where the target appears and instead program and execute a willful response to touch the opposite response box. Each of these subtasks is made up of 48 trials. Task is a within-subject independent variable and Group is a between-subject independent variable. The order of the PP and AP tasks was counterbalanced across subjects.

#### Procedure

As before, the dependent variable is RT, measured as the time from when the target is presented to when the subject initiates their response by lifting their finger from the center fixation point. All subjects were given the instructions and 4–6 practice trials before starting each task.

#### Data analysis

**Figure 7:**
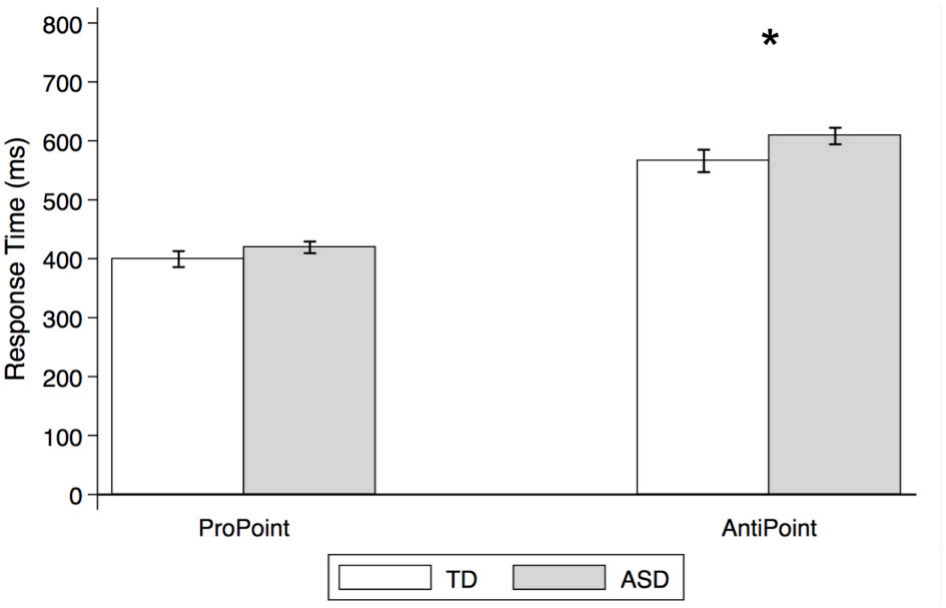
**Estimated mean response times for TD control and ASD children in non-social orienting tasks**. Response times (estimated means from mixed effect analysis, in ms) by TD control (white) and ASD (gray) subjects in ProPoint (reflexive) and AntiPoint (voluntary) non-social orienting tasks. A star indicates a significant group difference (^*^p < 0.05). Error bars represent one standard error.

A MATLAB script similar to the one used in Experiment 1 was used to extract data, trim errors and outliers, and calculate subject mean RTs used in the analysis. TD controls had an error rate of 3.23% (PP: 1.64%, AP: 4.76%), and ASD subjects had an error rate of 3.70% (PP: 1.73%, AP: 5.60%). Once these error trials were excluded, additional trials were filtered out from the final analysis according to the same criteria as in Experiment 1 except that the upper cutoff was set to 1200 ms to account for the longer RTs in the AP task. Consequently, for TD control and ASD subjects respectively, 5.07% (PP: 4.44%, AP: 5.69%) and 8.09% (PP: 5.61%, AP: 10.58%) of the remaining data were filtered out from the final RT analysis. As in Experiment 1, a mixed effect analysis was used to compare mean RT between groups for each task. In this analysis, Group (TD and ASD) is a fixed effect while Subject is a random effect with an autoregressive order 1 covariance structure. Estimated mean RTs from this analysis are presented in Figure 7.

### Results

As illustrated in Figure 7, there were no significant group differences in RTs in the PP task [*p* > 0.1; estimated mean RTs (± SE): 399.3 ms (±13.5) and 419.2 ms (±9.9) for TD and ASD, respectively]. In contrast, the ASD group is significantly slower than the TD control group in the AP task [*t*_(26)_ = 2.1, *p* = 0.036; estimated mean RTs (± SE): 566.0 (±19.0) ms and 608.1 ms (±14.1), for TD and ASD, respectively].

### Discussion

Our findings demonstrate no group differences on the ProPoint (PP) test of reflexive orienting but moderate differences on the AntiPoint (AP) test of voluntary orienting. Specifically, the ASD group took longer to respond in the AP task, which required that they inhibit their reflexive response to an illuminated target and instead press the opposite target. These results suggest deficits in non-social orienting and point to some overarching problems with voluntary orienting and its concomitant aspects of executive function in our sample of children with ASD.

It is possible that our ASD group was not uniformly impaired on our task of voluntary non-social orienting. Although our study was too small to further explore the issue of heterogeneity in our sample, there was no correlation between estimated IQs [Group mean (± SD): 102.3 (± 16.8)] and either PP or AP RTs for the ASD subjects (*p's* > 0.6*)*. In future research, it would be interesting to investigate in a larger ASD sample whether any factors such as IQ, verbal fluency, or specific subdiagnoses of ASD could predict whether voluntary orienting deficits are broad or specific to social orienting.

## General discussion

### Summary of main findings

In this study, we were able to isolate and define particular deficits in orienting behavior in a group of high-functioning autism spectrum disorder (ASD) children. First, we demonstrate intact reflexive social orienting in children with ASD. Furthermore, we found some evidence suggestive of enhanced reflexive social orienting in children with ASD compared to controls (non-predictive condition, long delay; Figure 3A). In contrast, these same ASD children demonstrated deficient voluntary social orienting compared to the TD Control group (Figure 5). Together, these findings show that there are important differences in reflexive and voluntary social orienting in children with ASD that may explain previous discrepancies in the literature and, more importantly, prove key in the development and targeting of strategies and interventions.

### Intact reflexive social orienting in ASD

First, our findings suggest that reflexive social orienting is preserved, or perhaps even enhanced, in the ASD group compared to TD controls. In the non-predictive (NP) condition, both ASD and control groups were significantly faster at congruent than incongruent trials at SOA of 523 ms and at the long delay (i.e., SOAs 523 and 783 ms combined) in the collapsed data. The reflexive cue effects were consistently greater in the ASD group than in the control group, although these group differences did not reach significance. Moreover, in the predictive (P) condition, children with ASD demonstrated a significantly greater facilitative reflexive cue effect than that of the control children at the short delay (i.e., SOAs 25 and 58 ms combined), raising the possibility of *enhanced* reflexive social orienting in children with ASD.

This evidence of intact reflexive social orienting is also consistent with the majority of previous studies investigating social orienting in ASD (Chawarska et al., 2003; Swettenham et al., 2003; Kylliäinen and Hietanen, 2004; Senju et al., 2004; Greene et al., 2011; Pruett et al., 2011). A few studies have reported deficient reflexive social orienting (Ristic et al., 2005; Goldberg et al., 2008; Gillespie-Lynch et al., 2013), but they differed from the current study in a number of important ways. First, Gillespie-Lynch et al. (2013) used a much younger sample (mean age = 4.75) and only used SOAs 400 ms and less in a NP task, which was earlier than we observed reflexive facilitation (at SOA of 523 ms). Further, both Goldberg et al. (2008) and Ristic et al. (2005) studies utilized line drawings of faces with eyes, which arguably reduces the social relevance of the stimuli (Hill et al., 2010). Other differences in methodologies, such as in how and for how long the gaze cue was presented and whether important comparisons were made between- or within-subjects, likely account for the discrepancies in these and other experiments using schematic cues (Ristic et al., 2005; Goldberg et al., 2008; Greene et al., 2011; Pruett et al., 2011).

### Deficient voluntary social orienting in ASD

Our ASD sample, despite being on the higher-functioning end of the spectrum and despite receiving explicit instructions about how a central gaze cue could help their performance, did not demonstrate voluntary social orienting using gaze cues. This lack of facilitation distinguished them from the control subjects, who became significantly faster at responding to targets cued by a predictive than a non-predictive gaze cue at SOA intervals that allowed sufficient time to process the direction of the gaze cue (at least 174 ms).

Our findings of impaired voluntary social orienting in ASD are important and differ in several respects from previous studies. First, we used a 100% predictive voluntary condition. Some previous studies have used “Predictive” conditions (Senju et al., 2004; Ristic et al., 2005; Pruett et al., 2011) where the gaze cues were only 80% predictive as compared to 100% predictive in our study. A more substantial difference that could account for discrepancies between studies with regards to whether voluntary orienting is intact or impaired may be due to confounded reflexive and voluntary processes. For instance, Pruett et al. (2011) and Ristic et al. (2005) reported intact voluntary social orienting in ASD in their 80% predictive conditions, but since the predicted trials were also the congruent trials, this design conflates voluntary with reflexive processes. Therefore, their lack of differences may be more indicative of intact *reflexive* rather than intact voluntary orienting in ASD. In contrast, Senju and colleagues, who did report differences in voluntary social orienting, used an 80% predictive condition in which the target appeared in the *opposite* direction from the gaze cue 80% of the time (i.e., 80% incongruent trials, 20% congruent), and this crucial difference, similar to our own study, may account for its differential findings from Pruett et al. (2011) and Ristic et al. (2005). In the present study, we compared a voluntary (100% predictive) condition to the exact same trials in a reflexive (50% predictive, i.e., non-predictive) condition. In this way, we were able to hold the congruency of the trials constant and look specifically at how subjects' response times differed when the gaze cue was 100% predictive from when it was non-predictive. However, findings from the Senju et al. (2004) study are perhaps most consistent with our own because their predictive condition did not conflate reflexive with voluntary processes. Specifically, they required the subjects to inhibit their reflexive response and instead look in the opposite direction of the gaze cues, separating voluntary from reflexive social orienting. In this way, such a predictive condition is more purely indicative of voluntary social orienting, which we, too, found to be impaired in ASD.

However, another important difference between our study and these previous studies is that in our tablet-based task, subjects' responses were measured as the time to lift their finger from the center fixation point (an initiation time, similar to Hill et al., 2010), moving their finger to the appropriate target, and touching the target. In contrast, these other studies have used key presses to measure reaction times (Senju et al., 2004; Ristic et al., 2005; Pruett et al., 2011), which include a movement component. Hence, in future work it would be interesting to examine whether there is a similar or different pattern of findings between groups, for example in movement duration or total (initiation plus duration) response times to touch the target.

### Non-social orienting is moderately impaired in our sample of ASD

Previous reports suggest children with ASD seem to be impaired on some measures of executive functions but not others (Liss et al., 2001; Geurts et al., 2004). Our results from Experiment 2 demonstrate impairments in executive function in our sample of high-functioning ASD children, as measured by our voluntary non-social orienting task. However, while interpreting these results, it is important to bear in mind the issue of heterogeneity within the autism population, especially when it comes to executive dysfunction (Liss et al., 2001). For instance, Kelly et al. (2013) found deficits on an antisaccade task in language-impaired children with and without an ASD diagnosis, but children with an ASD diagnosis but no language impairment did not show these same deficits. In contrast, in Experiment 2 of our study of high-functioning ASD children, we did observe significant differences between the TD control and ASD groups on voluntary but not reflexive non-social orienting tasks. It is possible that this group difference may have been driven by a subset of our ASD sample. The small size of and restricted range of language ability in our sample precluded us from directly investigating the relationship between language ability and general voluntary control. It is possible, therefore, that the differences we report in voluntary social orienting may be specific to *social* processes in some ASD subjects, but it is clear that some of our ASD subjects possess more general problems with voluntary control. Future studies are needed to examine and sort out the possibility and source of heterogeneity in ASD.

One promising avenue for future research on this topic would be to investigate how heterogeneity of ASD phenotypes relates to non-social voluntary orienting. Previous findings suggest that gaze-following accuracy is impaired and correlated with communicative skills in young children with autistic disorder but not in those with pervasive developmental disorder-not otherwise specified (PDD-NOS), a subtype of broader ASD under the previous DSM-IV criteria (Falck-Ytter et al., 2012). It is possible that more detailed measures of ASD diagnosis and/or particular deficits (such as in language) in a broader spectrum of ASD subjects may unearth important differences in executive functions as measured by the antisaccade/AntiPoint task within the ASD group (Kelly et al., 2013). Given the heterogeneity of the ASD population, this is an important question for the future development of therapies and training tools. Although our findings suggest that such interventions ought to primarily target voluntary social orienting, it is possible that some individuals would benefit from broader training in voluntary control.

## Conclusion

Overall, our findings provide a novel and valuable contribution to the understanding of social attention differences in ASD. Although children with high-functioning ASD were able to reflexively orient toward social gaze similarly to TD children, they did not exhibit any evidence of voluntary social orienting. In other words, when children with ASD are presented with a face with a potentially valuable social cue (i.e., averted gaze), their attention is automatically driven in the direction of that gaze as it is in TD children. However, we found that ASD children are not able to use this social cue in a voluntary, goal-directed, or context-dependent way, such as to facilitate response to a target when the gaze direction is 100% predictive of the target location. Future research should aim to further explore this difference and whether ASD individuals can be trained to use this information and overcome this deficit. However, the fact that predictive gaze cues are not facilitating these individuals' responses suggests that ASD individuals are not able to voluntarily use social gaze cues and thus may not find such cues particularly useful. This may help explain findings such as from Klin and colleagues that these individuals look at socially-relevant features, like eyes, significantly less than do typical individuals. In fact, one could even argue that failure to effectively use such information properly may train them to avoid the eyes and seek information elsewhere. Reduced eye contact in individuals with ASD may also be due to their especially strong emotional responses to eyes (Dalton et al., 2005), which could be in agreement with our finding of enhanced reflexive orienting in ASD. Our results are also consistent with the viewpoint that orienting to social cues is not universally impaired in ASD, but rather that deficits are specific and context-dependent (Chawarska et al., 2012; Guillon et al., 2014). In our view, different contexts will impose different demands on the voluntary control of social attention, and this will often result in observed social orienting deficits in ASD. Our findings that these individuals have the automatic ability to orient their attention using social stimuli, but are simply not doing so voluntarily, raise the exciting possibility of intervening and training this ability. For instance, tablet-based games similar to our social orienting iPad application may be an especially promising method of intervention. With these sorts of games, it may be possible to train these individuals to use social information to orient their attention in a more intentional, goal-directed manner. Since social orienting is important not only for everyday social interactions but also for other previously-described cognitive abilities like understanding others' actions, Theory of Mind development and even for typical language acquisition, such interventions may prove widely beneficial for children with ASD.

## Funding

This work was supported in part by National Eye Institute Vision Core Grant P30EY010608, a grant from Mission Connect, a program of TIRR Foundation, a Challenge Grant to The University of Texas Medical School at Houston from Research to Prevent Blindness, and the Hermann Eye Fund.

### Conflict of interest statement

The authors declare that the research was conducted in the absence of any commercial or financial relationships that could be construed as a potential conflict of interest. Co-authors A.B.S. and S.S.P. are named inventors in a patent application pending for a “Touch sensitive system and method for cognitive and behavioral testing and evaluation” (US 2014/0249447 A1).

## References

[B1] Akiyama T. Kato M. Muramatsu T. Saito F. Umeda S. Kashima H. (2006). Gaze but not arrows: a dissociative impairment after right superior temporal gyrus damage. Neuropsychologia 44, 1804–1810. 10.1016/j.neuropsychologia.2006.03.007 16616939

[B2] Castiello U. (2003). Understanding other people's actions: intention and attention. J. Exp. Psychol. Hum. Percept. Perform. 29, 416–30. 10.1037/0096-1523.29.2.416 12760625

[B3] Charman T. Baron-Cohen S. Swettenham J. Baird G. Cox A. Drew A. (2000). Testing joint attention, imitation, and play as infancy precursors to language and theory of mind. Cogn. Dev. 15, 481–498. 10.1016/S0885-2014(01)00037-5

[B4] Chawarska K. Klin A. Volkmar F. (2003). Automatic attention cueing through eye movement in 2-year-old children with autism. Child Dev. 74, 1108–1122. 10.1111/1467-8624.00595 12938707

[B5] Chawarska K. Macari S. Shic F. (2012). Context modulates attention to social scenes in toddlers with autism. J. Child Psychol. Psychiatry 53, 903–913. 10.1111/j.1469-7610.2012.02538.x 22428993 PMC3845814

[B6] Dalton K. M. Nacewicz B. M. Johnstone T. Schaefer H. S. Gernsbacher M. A. Goldsmith H. H.. (2005). Gaze fixation and the neural circuitry of face processing in autism. Nat. Neurosci. 8, 519–526. 10.1038/nn1421 15750588 PMC4337787

[B7] Dawson G. Meltzoff A. Osterling J. Rinaldi J. Brown E. (1998). Children with autism fail to orient to naturally occurring social stimuli. J. Autism Dev. Disord. 28, 479–485. 10.1023/A:1026043926488 9932234

[B8] Falck-Ytter T. Fernell E. Hedvall A. L. von Hofsten C. Gillberg C. (2012). Gaze performance in children with autism spectrum disorder when observing communicative actions. J. Autism Dev. Disord. 42, 2236–2245. 10.1007/s10803-012-1471-6 22354708

[B9] Friesen C. K. Kingstone A. (1998). The eyes have it! reflexive orienting is triggered by nonpredictive gaze. Psychon. Bull. Rev. 5, 490–495. 10.3758/BF03208827

[B10] Friesen C. K. Ristic J. Kingstone A. (2004). Attentional effects of counterpredictive gaze and arrow cues. J. Exp. Psychol. Hum. Percept. Perform. 30, 319–329. 10.1037/0096-1523.30.2.319 15053691

[B11] Frischen A. Bayliss A. P. Tipper S. P. (2007). Gaze cueing of attention. Psychol. Bull. 133, 694–724. 10.1037/0033-2909.133.4.694 17592962 PMC1950440

[B12] Geurts H. Verté S. Oosterlaan J. Roeyers H. Sergeant J. (2004). How specific are executive functioning deficits in attention deficit hyperactivity disorder and autism? J. Child Psychol. Psychiatry 45, 836–854. 10.1111/j.1469-7610.2004.00276.x 15056314

[B13] Gillespie-Lynch K. Elias R. Escudero P. Hutman T. Johnson S. P. (2013). Atypical gaze following in autism: a comparison of three potential mechanisms. J. Autism Dev. Disord. 43, 2779–2792. 10.1007/s10803-013-1818-7 23619947 PMC4066873

[B14] Goldberg M. C. Mostow A. J. Vecera S. P. Larson J. C. G. Mostofsky S. H. Mahone E. M.. (2008). Evidence for impairments in using static line drawings of eye gaze cues to orient visual-spatial attention in children with high functioning autism. J. Autism Dev. Disord. 38, 1405–1413. 10.1007/s10803-007-0506-x 18074212 PMC2693327

[B15] Greene D. J. Colich N. Iacoboni M. Zaidel E. Bookheimer S. Y. Dapretto M. (2011). Atypical neural networks for social orienting in autism spectrum disorders. Neuroimage 56, 354–362. 10.1016/j.neuroimage.2011.02.031 21334443 PMC3091391

[B16] Griffith E. M. Pennington B. F. Wehner E. A. Rogers S. J. (1999). Executive functions in young children with autism. Child Dev. 70, 817–832. 10.1111/1467-8624.00059 10446722

[B17] Guillon Q. Hadjikhani N. Baduel S. Rogeì B. (2014). Visual social attention in autism spectrum disorder: insights from eye tracking studies. Neurosci. Biobehav. Rev. 42, 279–297. 10.1016/j.neubiorev.2014.03.013 24694721

[B18] Hill E. L. (2004). Evaluating the theory of executive dysfunction in autism. Dev. Rev. 24, 189–233. 10.1016/j.dr.2004.01.001

[B19] Hill J. L. Patel S. Gu X. Seyedali N. S. Bachevalier J. Sereno A. B. (2010). Social orienting: reflexive versus voluntary control. Vision Res. 50, 2080–2092. 10.1016/j.visres.2010.07.020 20673778 PMC2956409

[B20] Kelly D. J. Walker R. Norbury C. F. (2013). Deficits in volitional oculomotor control align with language status in autism spectrum disorders. Dev. Sci. 16, 56–66. 10.1111/j.1467-7687.2012.01188.x 23278927

[B21] Kleinke C. L. (1986). Gaze and eye contact: a research review. Psychol. Bull. 100, 78–100. 10.1037/0033-2909.100.1.78 3526377

[B22] Klin A. Jones W. Schultz R. Volkmar F. Cohen D. (2002). Visual fixation patterns during viewing of naturalistic social situations as predictors of social competence in individuals with autism. Arch. Gen. Psychiatry 59, 809–816. 10.1001/archpsyc.59.9.809 12215080

[B23] Kylliäinen A. Hietanen J. K. (2004). Attention orienting by another's gaze direction in children with autism. J. Child Psychol. Psychiatry 45, 435–444. 10.1111/j.1469-7610.2004.00235.x 15055364

[B24] Landry O. Parker A. (2013). A meta-analysis of visual orienting in autism. Front. Hum. Neurosci. 7:833. 10.3389/fnhum.2013.00833 24367314 PMC3856368

[B25] Leekam S. Baron-Cohen S. Perrett D. Milders M. Brown S. (1997). Eye-direction detection: a dissociation between geometric and joint attention skills in autism. Brit. J. Dev. Psychol. 15, 77–95. 10.1111/j.2044-835X.1997.tb00726.x

[B26] Liss M. Fein D. Allen D. Dunn M. Feinstein C. Morris R.. (2001). Executive functioning in high-functioning children with autism. J. Child Psychol. Psychiatry 42, 261–270. 10.1111/1469-7610.00717 11280422

[B27] Morales M. Mundy P. Delgado C. E. F. Yale M. Messinger D. Neal R. (2000). Responding to joint attention across the 6- through 24-month age period and early language acquisition. J. Appl. Dev. Psychol. 21, 283–298. 10.1016/S0193-3973(99)00040-4

[B28] Munoz D. P. Everling S. (2004). Look away: the anti-saccade task and the voluntary control of eye movement. Nat. Rev. Neurosci. 5, 218–228. 10.1038/nrn1345 14976521

[B29] Nation K. Penny S. (2008). Sensitivity to eye gaze in autism: is it normal? Is it automatic? Is it social? Dev. Psychopathol. 20, 79–97. 10.1017/S0954579408000047 18211729

[B30] Posner M. I. Rafal R. D. Choate L. S. Vaughan J. (1985). Inhibition of return: neural basis and function. Cogn. Neuropsychol. 2, 211–228. 10.1080/02643298508252866

[B31] Pruett J. R. Jr. LaMacchia A. Hoertel S. Squire E. McVey K. Todd R. D.. (2011). Social and non-social cueing of visuospatial attention in autism and typical development. J. Autism Dev. Disord. 41, 715–731. 10.1007/s10803-010-1090-z 20809377 PMC3660145

[B32] Ristic J. Mottron L. Friesen C. K. Iarocci G. Burack J. A. Kingstone A. (2005). Eyes are special but not for everyone: the case of autism. Cogn. Brain Res. 24, 715–718. 10.1016/j.cogbrainres.2005.02.007 16099372

[B33] Senju A. Tojo Y. Dairoku H. Hasegawa T. (2004). Reflexive orienting in response to eye gaze and an arrow in children with and without autism. J. Child Psychol. Psychiatry 45, 445–458. 10.1111/j.1469-7610.2004.00236.x 15055365

[B34] Sereno A. B. (1992). Programming saccades: the role of attention, in Eye Movements and Visual Cognition, ed Rayner K. (New York, NY: Springer), 89–107.

[B35] Sereno A. B. Babin S. L. Hood A. J. Jeter C. B. (2009). Executive functions: eye movements and neuropsychiatric disorders, in Encyclopedia of Neuroscience, ed Squire L. R. (Oxford: Academic Press), 117–22.

[B36] Sereno A. B. Patel S. S. Shrestha Y. Red S. D. (2014). U.S. Patent Application Publication No. US 2014/0249447. Alexandria, VA: U.S. Patent and Trademark Office.

[B37] Swettenham J. Condie S. Campbell R. Milne E. Coleman M. (2003). Does the perception of moving eyes trigger reflexive visual orienting in autism? Philos. Trans. R. Soc. Lond. B. Biol. Sci. 358, 325–334. 10.1098/rstb.2002.1203 12639330 PMC1693118

[B38] Wechsler D. (1991). Wechsler Intelligence Scale for Children, 3rd Edn. San Antonio, TX: The Psychological Corporation.

[B39] Zhang M. R. Red S. D. Lin A. H. Patel S. S. Sereno A. B. (2013). Evidence of cognitive dysfunction after soccer playing with ball heading using a novel tablet-based approach. PLoS ONE 8:e57364. 10.1371/journal.pone.0057364 23460843 PMC3583826

